# Supplementary material for: Learning curve and surgical outcome of robotic assisted colorectal surgery with ERAS program
Source: Sci Rep. 2022 Nov 29;12:20566. doi: 10.1038/s41598-022-24665-w (PMC9709162; doi:10.1038/s41598-022-24665-w)
Supplement: Supplementary file 1 — Supplementary Information 1. [file 41598_2022_24665_MOESM1_ESM.zip › LSC Raw data-Ñ[▒K/149/H_ñw╝╨░O▒Kñσ.pdf]

正式摘要(電子簽章：000000234081445)

W102 / W102 ☐ 英文標題(注意:無英文姓名-請補病人基本檔!) ☐【讀取】憑證

臺中榮民總醫院

出院病歷摘要

轉入號 : 轉出號 :

[redacted]

**(9)**入院日期	111/01/05 (2022) 直腸外科 W102 - 191
**(10)**轉科日期	
**(11)**出院日期	111/01/09 (2022) 住院天數計 4 日

診斷

- 入院:1. A-Colon lesion with partial obstruction, r/o malignancy or Crohn's disease, with obstructive colitis
  2. Bloody stool and bowel partial obstruction with lumen narrowing (by 2020/12 colonscope), suspect tumor or Crohn's disease suggest survey but patient refuse (by 2020/10 abdominal CT as Focal asymmetrical wall thickening over hepatic flexure and proximal T-colon, underlying malignancy cannot be excluded)
  3. Cesarean section*2 years ago
  4. Unspecified neck myositis, under PO dexamethasone 4mg QW for 3-4 years
  5. Iron deficiency anemia, under IV iron injection Q2W
- 出院:1. Ascending colon leision suspect benign leision with partial obstruction and colitis, s/p Laparoscopic Right Hemicolectomy on 2022/01/06
  2. Bloody stool and bowel partial obstruction with lumen narrowing (by 2020/12 colonscope), suspect tumor or Crohn's disease suggest survey but patient refuse (by 2020/10 abdominal CT as Focal asymmetrical wall thickening over hepatic flexure and proximal T-colon, underlying malignancy cannot be excluded)
  3. Cesarean section*2 years ago
  4. Unspecified neck myositis, under PO dexamethasone 4mg QW for 3-4 years
  5. Iron deficiency anemia, under IV iron injection Q2W

主訴

- Abdomen RLQ pain for weeks

病史

- This is a 43 years old female patient with history of

  1. Colon lesion with partial obstruction, r/o malignancy or Crohn's disease, with obstructive colitis

  2. Bloody stool and bowel partial obstruction with lumen narrowing (by 2020/12 colonscope), suspect tumor or Crohn's disease suggest survey but patient refuse (by 2020/10 abdominal CT as Focal asymmetrical wall thickening over hepatic flexure and proximal T-colon, underlying malignancy cannot be excluded)

  3. Cesarean section*2 years ago

  4. Unspecified neck myositis, under PO dexamethasone 4mg QW for 3-4 years

  5. Iron deficiency anemia, under IV iron injection Q2W

  According to the previous medical record, she had abdomen RLQ pain on 2021/12/19 night and she visited FongYuan H. ER, where abdominal CT was done showed ileus, R/I malignancy obstruction. Then she was transfered to our ER and admitted to our GI ward on 12/20 for further survey and management. After Antibiotic treatment and supportive care, she felt symptoms relived and AAD on 12/23. She visited our CRS OPD on 12/29. After discussion, she prefered surgical intervention. This time she was admitted for scheduled operation.

體檢發現

-
  - General appearance :
    - easy looking , conscious clear , without cardiopulmonary distress
    - Vital signs自動帶入date 體溫(BT) 脈搏(P) 呼吸(R) 血壓(BP) 身高(BH) 體重(BW) BMI
      20220105 36C 90/min 16/min 136/55mmHg 159cm 60kg 23.7
    - Vital signs: B.P. 136/55 mmHg , P.R. 90 /min , R.R. 16 /min , B.T. 36 ℃
  - HEENT :
    - Eyes: no anemic conjunctiva, no icteric sclera, normal EOM

-
    - ENT: no otorrhea, no rhinorrhea, no erythematous mucosa, no injected tonsil, no deformity
- Neck: supple, freely movable, trachea midline,no deformity, no engorged jugular vein , no palpable thyroid or lymph node enlargement
- Chest:
    - Inspection: Grossly normal, symmetric and fully expansion, no accessory muscle used, no operation scar, no spider angiomata, no gynecomastia
    - Palpation: no palpable mass or nodule, no subcutaneous emphysema, no tenderness
    - Ausculation: clear breathing sound
- Heart:
    - Percussion: normal heart size
    - Ausculation: regular heart beat, normal S1, S2, no gallop, no murmur
- Abdomen:
    - Inspection: in
        - flat contour, symetrically, no scar, no stria, no dilated veins, no rashes
    - Ausculation: normoactive bowel sound, no abdominal bruits
    - palpation: soft, no muscle guarding or rigidity, no local tenderness, no rebounding pain, no palpable mass, no palpable pulsation
    - Percussion: no shifting dulllness, no hepatomegaly, no splenomagaly
-

Peripheral pulsation :	CA	BA	RA	FA	PA	PTA	DPA
Right side :	++	++	++	++	++	++	++/+++
Left side :	++	++	++	++	++	++	++/+++

- Genito-anus: no tenderness or palpable mass lesion or rectal shelf, normal external genitalia, normal anal tone, brown stool

CRS CONDITION

- CEA: 0.84 ng/ml
-
    - Lung

手術日期及方法

- 手術日期及方法20220106 Laparoscopic right colectomy and anastomosis
- 手術結果及發現 :
    - 手術日期: 1110106 麻醉方式:
      手術名稱:Laparoscopic right colectomy and anastomosis
      術前診斷:RO Malignant tumor of A-colon
      術後診斷: Ascending colon leision suspect benign leision s/p laparoscopic right hemicolectomy

住院治療經過

- After admission, she recieved Laparoscopic RH on 2022/01/06. She strat try Bland II diet on 1/08 and tolerate well. Under relative stable condition, she discharged on 2022/01/09, arrange OPD follow up.

合併症

一般檢查

- 累積報告 :

DATE	TIME	ALB	TP	BIL.T	BIL.D	ALKP	AST	ALT	LDH	AMY	LIPASE	NH3	Ferritin	UA
1101220	1249	3.3	6.3	0.5	0.1	67	21	18	202	42	10			
1101221	1408			0.49		73		17	190				123.70	
1101221	1426										13			
1110105	2208	3.4	6.0	0.27	0.06	60	14	11						3.6
1110106	1650	3.0		0.30	0.09	57	15	9						

DATE	TIME	WBC	RBC	HGB	HCT	MCV	PLT	BLST	NEUT	BAND	SEG	LYM	MON	EOS	BAS
1101220	1249	1840	4.05	9.7	33.0	81.5	404				74.0	16.0	10.0	0.0	0.0
1101221	1409	6870		9.3			387				85.0	3.0	12.0	0.0	0.0
1110105	1945	7270	3.39	8.4	28.1	82.9	453		75.3			15.5	6.7	1.8	0.7
1110106	1649	11140	4.25	10.3	34.1	80.2	433		89.9			5.7	3.7	0.3	0.4

DATE	TIME	NA	K	CL	BUN	CREAT	eGFR	CA	Mg	P	CRP	ESR	PCT	D-Dimer	LACTATE
1101220	1249	139	3.5		19	0.90	72.63	7.6	2.2	2.2	3.913				
1101221	1400													2.83	
1101221	1408	135	4.4		11	0.82	80.87	8.2	2.2						
1101221	1410											36			
1110105	2208	137	3.7		17	0.93	69.93	8.1			1.036				
1110106	1650	141	3.7	109	10	0.72	93.96	7.7							

DATE	TIME	GLUCOSE	HbA1C	CHOL	TG	HDL	LDL	KETONE	CK	CKMB	TRO-T	PT-P	APTT-P	NT-pro BNP
1101220	1248											10.5	31.3	

-

1101220	1249								58	18	5.91			220.20
1101221	1408								39					
1101221	1409	103												
1101221	1425							0.4						
1110105	1943		5.1											
1110105	1946	75												
1110105	2208			184	86	56	115							
1110106	1649	97												

DATE	TIME	SP-GR	PH	PROT	GLUC	KETON	BILI	UROB	OB	NITR	LEU	RBC	WBC	EPCEL
1101220	1420	1.029	7.0	Trace	Negative	1+	Negative	0.2	Trace	Negative	Negative	5-10	0-2	2-4
1110108	1621	1.018	7.5	Negative	Negative	1+	Negative	0.2	Negative	Negative	Negative	0-2	0-2	6-10

DATE	TIME	PH	BEB	SCVO2	PO2	HCO3	HGB	PCO2	TCO2
1101220	1247	7.412	0.6	85.2	49.4	25.2		40.6	26.5
1101221	1356	7.361	-0.5	35.4	22.3	25.1		45.3	26.5

特殊檢查

病理報告

其它

出院時情況

- 3.改門診治療

出院指示

-

藥名	劑量	劑量單位	用法	頻次	數量	數量單位	現況	天數	單位
Ultracet tab	1	TAB	PO	Q6H	44	TAB	TH	11	天
(□)Takepron OD tab 30mg	1	TAB	PO	QDAC	11	TAB	TH	11	天
Chlorzoxazone-250	1	TAB	PO	TID	33	TAB	TH	11	天
Mag. Oxide tab	2	TAB	PO	TID	66	TAB	TH	11	天

出院計劃

- 門診日期
  - date on 2022/01/19

主治醫師 2062F 蔣鋒帆 / 住院醫師 2339C 蔡濬樟

正式摘要(電子簽章：000000234081445)

W102 / W102

doctorRank:B
